# Utility of Equivalence and Cognition Models in Enhancing Validity of Translated Questionnaires: A Methodological Example Using the Heart Quality-of-Life Questionnaire

**DOI:** 10.1097/jnr.0000000000000414

**Published:** 2020-12-07

**Authors:** Wan Ling LEE, Khatijah LIM ABDULLAH, Karuthan CHINNA, Imran Z. ABIDIN

**Affiliations:** 1 PhD, RN, Senior Lecturer, Faculty of Medicine, Department of Nursing Science, University of Malaya, Kuala Lumpur, Malaysia; 2 DClinP, RN, Professor, Department of Nursing, School of Healthcare and Medical Sciences, Sunway University, Kuala Lumpur, Malaysia; 3 PhD, Associate Professor, Faculty of Health and Medical Sciences, School of Medicine, Taylor's University, Malaysia; 4 MMed, MBBS, Professor, Faculty of Medicine, Department of Medicine, University of Malaya, Kuala Lumpur, Malaysia.

**Keywords:** coronary nursing, translations, health-related quality of life, patient-reported outcome measures, cognitive interviewing

## Abstract

**Background:**

The cross-cultural adaptation of questionnaires has tenuous theoretical underpinnings that limit the rigor of data collection and the meaningful analysis of cognitive interview data. An adaptation of existing models of equivalence and cognition provides structure to the comprehensive investigation of various equivalence types in enhancing the validity of translated questionnaires.

**Purpose:**

In this study, a framework comprising equivalence and cognition models was used to assess and finalize the Heart Quality-of-Life (HeartQoL)-Bahasa Malaysia (BM) questionnaire, which was derived from both forward–backward (FB) and dual-panel (DP) translation methods.

**Methods:**

Investigation and finalization of two initial versions of the questionnaire were conducted based on findings from an expert assessment (*n* = 3 sociolinguists blinded to translation methods) and cognitive interviews with purposively sampled patients (FB: *n* = 11; DP: *n* = 11). The equivalence model of Herdman et al. and the question-and-answer model of Collins were adapted to form a “cognition-and-equivalence” model to guide data collection and analysis through modified cognitive interviews. The final HeartQoL-BM was completed by 373 patients with ischemic heart disease from two medical centers, and the data were analyzed using confirmatory factor analysis to assess the evidence of equivalence.

**Results:**

Findings from the expert assessment and cognitive interview showed the existence of semantic and item equivalence on almost all of the FB and DP items, identified some subtle potential equivalence gaps, and guided the process of item finalization. Confirmatory factor analysis, including tests of factorial invariance on the final two-factor model of HeartQoL-BM, confirmed conceptual, item, measurement, and operational equivalence, which supports functional equivalence.

**Conclusions:**

The potential use of the cognition-and-equivalence model for modified cognitive interviewing and the application of the six equivalence types of Herdman et al. were supported by the HeartQoL-BM showing functional equivalence with its source. HeartQoL-BM is a potentially valid measure of health-related quality of life for patients with ischemic heart disease independent of conditions such as angina, myocardial infarction, and heart failure.

## Introduction

An adapted questionnaire (also known as a target or translated version) is assumed to be comparable with the original (or source) questionnaire after equivalence between the two versions has been established. Findings based on the equivalent target version are taken as valid, which allows data pooling and interstudy comparisons to be made across cultures. The methods used to test equivalence vary, and current empirical evidence is not adequate to pool consensus. Moreover, no gold standard exists to define the degree of acceptable similarities or tolerable variance to conclude equivalence between target and source versions. Experts, in general, agree on a multistep process for the cross-cultural adaptation of questionnaires (Eremenco et al., 2017). Most experts support using cognitive interviews to examine comparability between versions by exploring respondents' thought processes, especially with regard to their interpretation of item meanings and relevance. The wide-ranging, cross-cultural application of questionnaires with no consensus on analysis approach is the Achilles' heel of cognitive interviewing (Willis, 2015a). Thus, research advancing an appropriate analysis model is warranted. Research on the theoretical basis and the methodology necessary to establish equivalence is lacking, and the number of studies examining issues surrounding the cross-cultural adaptation of questionnaires has declined in recent years. Researchers are more explicit in reporting psychometrics properties (i.e., measurement equivalence) than other types of equivalence. To establish the many types of equivalence, both qualitative and quantitative approaches are needed, as no single method is able to address all equivalence types.

### Background

This study sought to adapt the equivalence model of Herdman et al. (1998) and the “question-and-answer” cognition model of Collins (2003) as part of a larger study that was designed to establish six types of equivalence (conceptual, item, semantic, operational, measurement, and functional) in the Malay version of the Heart Quality-of-Life (HeartQoL) scale. According to Herdman et al., “conceptual equivalence” exists if the adapted questionnaire has the same relationship to the underlying concept (i.e., factors) between source and target culture. “Item equivalence” is the extent to which items are relevant and acceptable across cultures. This term also refers to the extent to which items estimate the same parameters of factor structures. “Semantic equivalence” refers to the transfer of item meaning to achieve a similar effect on respondents in different languages. “Operational equivalence” exists when a similar format, instructions, mode of administration, and measurement methods produce comparable results between adapted and source questionnaires. “Measurement equivalence” is the extent to which the properties of the measurements in the adapted questionnaire approximate its source. “Functional equivalence” (aka the summary of equivalence), which refers to the extent to which an instrument performs equally well across cultures, may be established only after the other five equivalence types have been substantiated. A framework mapping the six equivalence types to the complete process of cross-cultural adaptation of questionnaire has been proposed (W. L. Lee et al., 2019). However, the concepts of equivalence and how they link to the cognition processes underpinning cognitive interview remain underexplored in the literature. Thus, this study was designed to integrate equivalence into a cognition model to propose a structured guide for data collection and analysis of cognitive interviews employed in pretesting of translated questionnaires.

The methodologies used to translate questionnaire are still largely opinion based, with existing evidence insufficient to verify conclusively the value of back-translation, which currently dominates the field (Colina et al., 2017; Epstein et al., 2015). Therefore, in this study, the HeartQoL scale was translated into Malay (Bahasa Malaysia or BM) using two popular translation methods, the forward–backward (FB) method and the dual-panel (DP) method, as described respectively by Beaton et al. (2000) and Swaine-Verdier et al. (2004). The methodology used to finalize and validate the synthesized HeartQoL-BM to establish the six types of equivalence is described in this article.

### Heart Quality-of-Life Questionnaire

The copyrighted, 14-item HeartQoL is a core measure of health-related quality of life (HRQoL) for patients with ischemic heart disease (IHD) that is independent of myocardial infarction, angina, and heart failure conditions. The participants were asked to rate on a scale of 0 (*none*) to 3 (*a lot*) regarding how much they felt bothered when in specific situations or while doing specific tasks. The detailed content of the HeartQoL is available elsewhere (Oldridge et al., 2014). Although the test–retest reliability of this scale has been verified in Malaysia (W. L. Lee et al., 2016), its construct validity has been established predominantly in Western populations (De Smedt et al., 2016; Oldridge et al., 2014). Thus, qualitative inquiry is deemed necessary to assess HeartQoL equivalence from an East Asian perspective.

## Methods

Ethics clearance to conduct this study at two medical centers in Kuala Lumpur (reference: No 996.45 and RD5/25/14) and permission to translate the HeartQoL into BM were obtained. The preliminary conceptual and item equivalences of the original HeartQoL were verified by two local cardiologists and five English-speaking patients as recommended in Herdman et al. (1998). Because of the high level of semantic similarity between the FB and DP versions (W. L. Lee et al., 2019), further examination was conducted to assess the equivalence of each version with the source questionnaire using expert assessment and cognitive interviews to aid decision making in item finalization for a synthesized HeartQoL-BM. Afterward, the finalized HeartQoL-BM was validated using a cross-sectional survey.

Details on the original model of equivalence (Herdman et al., 1998) and the question-and-answer model (Collins, 2003) are provided elsewhere. This article was designed to affirm the semantic and item equivalences by analyzing cognitive interviews using the proposed cognition-and-equivalence model. Evidence of conceptual, item, operational, and measurement equivalences were verified using a confirmatory factor analysis of the HeartQoL-BM, which was administered in a survey. Functional equivalence was confirmed when the five equivalence types were substantiated, which, in turn, supported the workability of the proposed framework.

The following sections describe the data collection and analysis approaches used in the expert assessment, cognitive interviews, and survey, which were conducted between December 2015 and April 2016. Expert assessment was performed by sociolinguists from three different universities. All of these individuals were native BM speakers, proficient in English, and experienced translators. The patients who participated in the cognitive interview and survey proceedings were recruited from cardiac wards if they had an indexed diagnosis of IHD, were > 18 years old, and spoke BM as a first or second language. Patients who were cognitively unfit or hemodynamically unstable were excluded.

### Expert Assessment

This method produced grand means and corresponding comments on semantic equivalence. The findings were triangulated with the cognitive interviews to aid item finalization. Three bilingual sociolinguists who were blinded to the translation process performed independent assessments of the FB and DP versions. Detailed ratings across three aspects of semantic equivalence are available elsewhere (W. L. Lee et al., 2019). In this study, ratings were averaged to compute a grand mean (X_GM_) value ranging from 0 (*very poor*) to 6 (*excellent*). An X_GM_ of 4.0–6.0 was taken as an indicator of good-to-excellent overall semantic equivalence (Keller et al., 1998), with a higher X_GM_ value indicating closer semantic equivalence of the item version to its source.

### Cognitive Interview

In assessing semantic and item equivalences in terms of item content relevance, the proposed cognition-and-equivalence model was applied to guide the data collection and analysis of the cognitive interview process. Eligible patients were selected using purposive parallel quota sampling (Collins & Gray, 2015) to acquire a sample that was representative across the following demographics: (a) male and female gender; (b) primary, secondary, and tertiary levels of education; (c) Malay and non-Malay ethnicity; and (d) age groups (< 50, 50–60, and > 60 years). Sampling ceased at data saturation, and sample sizes were equal for both FB and DP versions. As the copyright use agreement prohibited the modification or deletion of items, it was pragmatic to employ a modified cognitive interview process that focused on examining items primarily for semantic and item equivalence.

**Figure 1.**
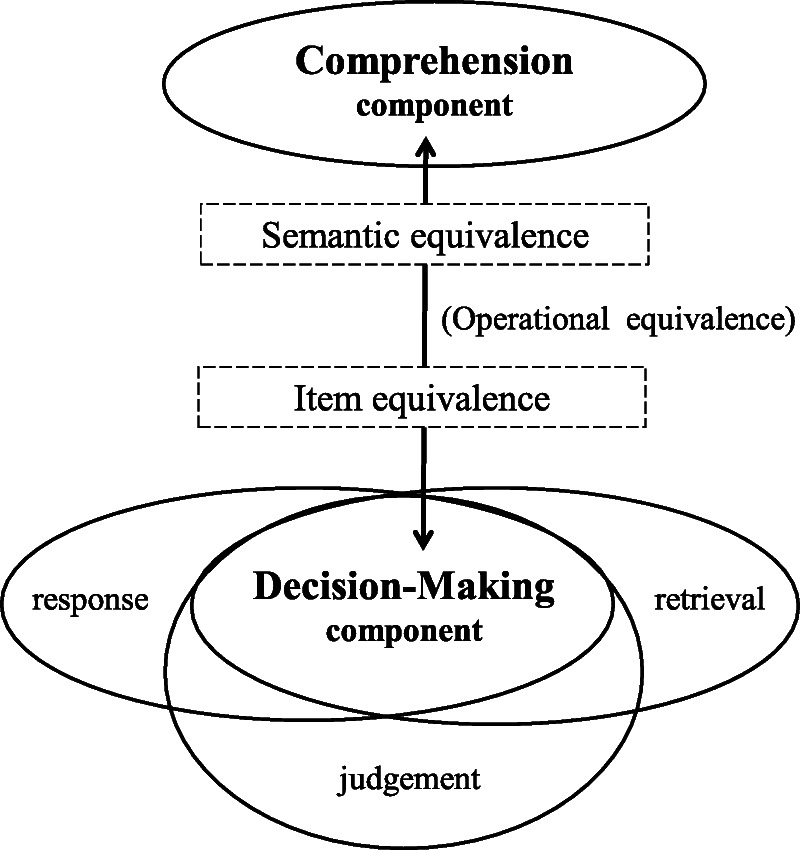
The “cognition-and-equivalence” model used to guide the cognitive interviews was adapted from the question-and-answer model (Collins, 2003) and the equivalence model (Herdman et al., 1998)

Collins' (2003) question-and-answer model, which underpins Tourangeau's four-stage cognition, was adapted in this study to guide the equivalence investigation (via the cognition-and-equivalence model). In Figure 1, the two-headed arrow linking the “comprehension*”* and “decision-making*”* components of cognition represents the interdependency and iterative interaction of these two components. The “decision-making” component represents the complex, intertwined interactions between the retrieval, judgment, and response processes of cognition, which help respondents determine their answer.

#### Assumptions of the cognition-and-equivalence model

The evidence for “semantic equivalence” and “item equivalence” were extracted by exploring

(a) patients' comprehension, that is, whether each item was understood or interpreted as intended by the original item using questions such as “Can you explain the meaning of this sentence (or this term ‘X’)?” and “Please give examples to explain its meaning”; and
(b) patients' decision making, that is, whether the item was perceived as relevant and appropriate to their setting by eliciting the reasoning used by the participants to determine their answers. Example questions include “Why did you choose that particular answer?”, “Is it suitable to ask this question? Why?”, and “Why is the question difficult or easy to answer?”

In addition, participant feedback was solicited regarding the clarity of instructions and response options, the practicality of the format layout, and the 4-week recall period using questions such as “Give an example of the timeframe you are thinking of when answering?” (referring to a 4-week recall), “What do you think of the wording size and layout?”, and “Which part of the questionnaire do you not like?” However, this approach is adequate only to support “preliminary operational equivalence.” According to Herdman et al. (1998), full operational equivalence should be established based on findings related to measurement properties during questionnaire validation.

The participants were interviewed after self-administering either the FB or DP version. The interview sessions were kept within 30 minutes, as the participants get tired easily. The interviews were generally audio-recorded. Alternatively, interviewer notes were used when participants declined permission to be audio-recorded. About two interviews were conducted per day, and the data were analyzed on the same day. Scripted probes were revised accordingly to suit Malaysian patients, especially those with less education, with lower survey literacy, and of higher ages, who were likely to be relatively reticent, less accustomed to research culture, and less articulate than their peers in other cultural settings (Park et al., 2016). To ensure consistency, the same interviewer and coresearch team analyzed the data and performed peer-checking. Descriptive analyses of the data were performed using a text-summary approach (Willis, 2015a) to confirm semantic, item, and preliminary operational equivalences and identify significant problems in drawing conclusions. The findings were summarized in a matrix framework based on the primary objectives of the modified cognitive interview process. According to Willis (2015b), the detection of flaws and resultant decisions depend on the judgment of researchers instead of the opinions of participants, as the latter are not generally regarded as “experts” in cognitive interview proceedings. Findings on the corresponding FB and DP items were compared to aid decision making in finalizing the HeartQoL-BM.

### Survey

A survey was used to generate the responses for the confirmatory factor analysis (CFA), which assessed the HeartQoL-BM in terms of conceptual, item, operational, and measurement equivalences. To assess HeartQoL-BM across the three IHD conditions, eligible patients were consecutively sampled to recruit a minimum of 100 participants from each one of the three subgroups (Kline, 2005): patients with a myocardial infarction between 1 and 6 months, patients treated mainly for angina, and patients who had ischaemic heart failure. Data from the 4-point HeartQoL were treated as ordered categorical data to generate polychoric correlation matrices for CFA using EQS Version 6.3 (Multivariate Software, Inc., Temple, CA, USA). The nonnormal data were analyzed using a robust (or corrected) maximum likelihood estimator to provide adjusted standard errors and Satorra–Bentler scaled chi-square statistics. The acceptability of overall model fit was based on Hu and Bentler's (1999) recommendations, whereas the Fornell and Larcker (1981) model was used to test the discriminant validity of the model-based factors. The HeartQoL-BM was tested equivalently across the subgroups for measurement invariance. Goodness-of-fit may be confirmed only when the *p* value of the corrected Satorra–Bentler's χ^2^ difference is > .05 (Satorra & Bentler, 2010) and the comparative fit index–robust difference is ≤ 0.01 (Byrne, 2006).

### Ethical Considerations

Informed consent was obtained from all of the participants, and all procedures were undertaken in accordance with the ethical principles outlined in the Declaration of Helsinki.

## Results

In comparing the corresponding items in the FB and DP versions, four item pairs (HQ [HeartQoL item] 1, HQ2, HQ6, and HQ14) were found to be nonidentical (28.6%), whereas the remaining items were identical or almost identical in wording. All item versions had good overall semantic equivalence (X_GM_ ≥ 4.0) with the exception of HQ6_FBversion_, HQ9_FBversion,_ HQ9_DPversion_, and HQ14_DPversion_ (X_GM_ = 2.55–3.83). The final HeartQoL-BM included the item versions that earned the highest grand mean scores, while taking into consideration the findings of cognitive interviews. The resultant HeartQoL-BM was composed of six DP items (HQ_DPversion_ 1, 2, 4, 6, 10, and 13), four FB items (HQ_FBversion_ 3, 8, 9, and 14), and four identical items. A sample detailing the findings of the expert assessment and cognitive interviews that guided item finalization in HeartQoL-BM is shown in Table 1.

**Table 1.** Selected Examples of Findings—Summary of Flagged Items of HeartQoL-BM and Its Corresponding Item Finalization

Expert Assessment (*n* = 3)	Cognitive Interview (FB: *n* = 11; DP: *n* = 11)	Item Finalization
**Items with potential gaps in semantic equivalence**		
HQ7 (being physically restricted): X_GM_ = 5.06 (FB = DP) Experts A and C commented the term “fizikal” (physical) sounded technical.	Two patients with low education could not define the term “fizikal” (e.g., Patients d and i).	Although HQ7 was identical between the FB and DP versions, the semantic equivalence of item was enhanced with “anggota badan” added to cue the meaning of “fizikal.”
HQ10 (feeling depressed): X_GM_ = 4.22_FB_ vs. 5.22_DP_ All experts suggested the use of “depress” rather than “murung.”	Nonnative Bahasa-Malaysia-speaking patients were not familiar with “murung” (e.g., Patients B and d).	HQ10_DPversion_ with added synonyms, i.e., “murung (depression/sangat sedih/hilang semangat hidup)” was accepted as it had higher scores.
Items with potential gaps in item equivalence		
HQ2 (gardening/vacuum/carry groceries):X_GM_ = 4.95_FB_ vs. 4.28_DP_ Experts B and C preferred “barangan dapur” than “barangan runcit” to describe groceries. All experts commented that sweeping and mopping were more common than vacuuming as a cleaning chore in typical Malaysian households.	Tasks in HQ2 were valued differently because of patriarchal gender-role, e.g., womenfolk doing household chores most of the times. Sixteen of 22 patients swept and mopped the floor more frequently than vacuuming.	Although HQ2_DPversion_ had a slightly lower X_GM_, it was selected for its culturally relevant examples. The final HQ2 had sweeping/mopping added to the original item.

***Note*.** Bahasa Malaysia is also known as the Malay language; words in quotation marks are of Malay language. HeartQoL-BM = Heart Quality-of-Life-Bahasa Malaysia questionnair; HQ = HeartQoL item; FB = forward–backward; DP = dual-panel; X_GM_ = mean of ratings from three experts.

### Cognitive Interviews and Expert Assessment

The patient subgroups (*N* = 22) included in the cognitive interview process were, by FB–DP ratio, (a) male (8:7) and female (3:4); (b) Malays (6:7), Chinese (2:1), and Indians (3:3); (c) 25–49 years old (4:2), 50–59 years old (4:5), and 60–77 years old (3:4); and (d) primary (3:1), secondary (5:7), and tertiary (3:3) education. The expert feedback was congruent with the findings from the cognitive interviews. Selected examples of flagged items with potential gaps of semantic and item equivalences are shown in Table 1. In the final version, item versions with relatively higher grand means were selected over their equivalent, lower-grand-mean versions unless shortcomings were identified during the cognitive interview process.

All of the participants were able to read out the texts and mark their responses on paper. Similar findings were identified across FB and DP subgroups. Some participants (e.g., Patients H and k) described their mental retrieval of memory on a whole-month rather than week-to-week basis in recalling a 4-week event or experience. Some participants (e.g., Patients F, G, and g) preferred to estimate their degree of bother by recalling the frequency rather than the intensity of symptoms unless these symptoms were severe. Moreover, both participants and experts expressed difficulty in quantifying the response option “somewhat bothered,” as there is no equivalent expression in BM. As some participants (e.g., Participant B) commented on having a natural inclination to associate “none” with a score of 0, the final HeartQoL-BM scale was scored in ascending order, with 0 = *not bothered at all* and 3 = *greatly bothered*. These responses were reverse coded before computing scores. These observations shed light on the value of cognitive methods in creating awareness of the complexity surrounding the cross-cultural adaptation of questionnaires. However, it is beyond the scope of this article to elaborate on this issue.

### Factor Structure of the Final Heart Quality-of-Life–Bahasa Malaysia

Responses from 373 patients were used in factor structure testing. As shown in Table 2, no significant difference in the distribution of patients' characteristics across the three IHD conditions was found. Moreover, the ceiling effect was evident in the emotional subscale for patients with angina and those with myocardial infarction. Table 3 illustrates a summary of the assessments of various models of HeartQoL-BM. The three-factor model (Model C) lacked discriminant validity, as F2 (physical functioning) was highly correlated with F3 (physical symptoms) at *r* of .93. Hence, the popular Wilson–Cleary's HRQoL model was not supported. The two-factor model (Model B) showed acceptable fit indices with sufficient discriminant validity, with all factor loadings > .80. The second-order factor structure model (Model B.1) showed good model fit, with factor loadings ranging from .64 to .98. As shown in Table 4, Model B had sufficient factorial invariance across the subgroups of myocardial infarction, angina, and heart failure as well as across native and nonnative BM speakers.

**Table 2.** Distribution of Patient Characteristics and Floor Ceiling Effects, by Total Group and Subgroups in the Survey

	Myocardial Infarction (*n* = 123)		Angina (*n* = 139)		Heart Failure (*n* = 111)		Total Group (*N* = 373)	
Patient Subgroup and Characteristic^a^	*n*	%	*n*	%	*n*	%	*n*	%
Male gender	104	84.6	113	81.3	98	88.3	315	84.5
Married	116	94.3	134	96.4	100	90.1	350	93.8
Ethnicity ^b^								
Malay	73	59.3	62	44.6	55	49.5	190	50.9
Chinese	12	9.8	30	21.6	23	20.7	65	17.5
Indian	36	29.3	43	30.9	31	27.9	110	29.5
Others	2	1.6	4	2.9	2	1.8	8	2.1
Educational level								
Tertiary	30	24.4	47	33.8	25	22.5	102	27.3
Secondary	78	63.4	79	56.8	76	68.5	233	62.5
Primary	15	12.2	13	9.4	10	9.0	38	10.2
Employment status								
Employment	51	41.5	51	36.7	27	24.6	129	34.6
Retiree	47	38.2	57	41.0	46	41.8	150	40.2
Others	25	20.3	31	22.3	38	34.6	94	25.2
Diabetes mellitus	65	52.8	78	56.1	73	66.4	225	60.3
Hypertension	95	77.2	110	79.1	83	75.5	288	77.2
BMI > 30	2	1.6	12	8.6	6	5.5	20	5.4
Active smokers	32	26.0	17	12.2	13	11.8	63	16.9
Age (years; mean)	59.5	*SD =* 10.6	61	IQR = 12.0	58.9	*SD* = 13.4	60.0	IQR = 14.0
HeartQoL-BM version (score range: 0–3)								
Physical subscale (mean scoring of Items HQ1–HQ8, HQ13, and HQ14)								
Ceiling effect ^c^	1	0.8	3	2.2	1	0.9	5	1.3
Floor effect ^d^	0	0.0	0	0.0	2	1.8	2	0.5
Emotion subscale (mean of scoring Items HQ9–HQ12)								
Ceiling effect ^c^	33	26.8	45	32.4	3	2.7	106	28.4
Floor effect ^d^	2	1.6	0	0.0	6	5.5	8	2.1
Global subscale (mean of scoring all items HQ1–HQ14)								
Ceiling effect ^c^	0	0.0	2	1.4	0	0.0	2	0.5
Floor effect ^d^	0	0.0	0	0.0	1	0.9	1	0.3

***Note*.** BMI = body mass index; IQR = interquartile range; HeartQOL-BM = Heart Quality-of-Life-Bahasa Malaysia questionnaire; HQ = HeartQoL item.

^a^ No significant difference (*p* > .05) in age, gender, marital status, ethnicity, educational level, diabetes, and hypertension status across subgroups. ^b^ Patients of Malay ethnicity are native BM speakers, whereas the rest of ethnic groups are nonnative BM speakers. ^c^ Number of patients with a maximum mean score of 3. ^d^ Number of patients with a minimum mean score of 0.

**Table 3.** Structure of the HeartQoL-BM: Summary of Model Assessment in Total or Single-Group Analyses (*N* = 373)

Model		Goodness of Fit							Factor-Based Reliability and Validity			
No.	Descriptions	*df* (Cutoff criteria)	SBχ^2^	TLI_robust_	CFI_robust_	RMSEA_robust_/90% CI (< .06 = good fit ^a^; > .10 = poor fit ^b^)	CAIC_robust_		CR (> .7)	AVE (> .5)	MSV (< AVE)	ASV (< AVE]
				(≥ .95 = good fit ^a^)								
First-order, one-factor Model A									–	–	–	–
A	**F**: HQ1–HQ14	77	757.17	.956	.962	.154 [.144, .164]	224.21					
First-order, two-factor Model B ^c^												
B	**F1** (emotion): HQ9–HQ12	76	150.82	.995	.996	.051 [.039, .063]	−375.22	F1	.843	.826	.407	.407
	**F2** (physical): HQ1–HQ8, HQ13–HQ14							F2	.938	.722	.407	.407
Source results ^d^				.94	.95	.117						
First-order, three-factor Model C—based on Wilson and Cleary's conceptual model and exploratory confirmatory factory analysis												
C	**F1** (emotion): HQ9–HQ12	73	99.3	.998	.999	.031 [.012, .046]	−405.97	F1	.843	.827	.473	.404
	**F2** (physical functioning): HQ1–HQ5, HQ13							F2	.881	.661	.859 ^e^	.597
	**F3** (physical symptoms): HQ6–HQ8, HQ14, HQ13							F3	.860	.631	.859 ^e^	.666
Second-order, two-factor Model B.1 ^f^												
B.1	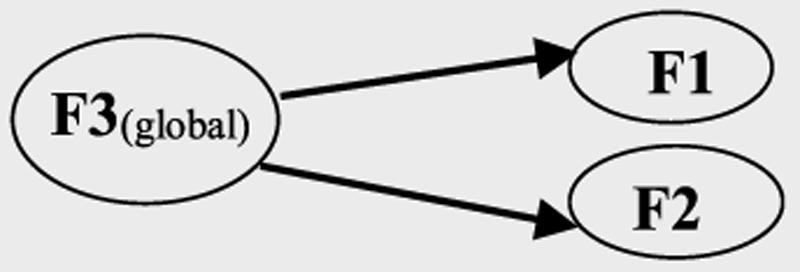	75	148.70	.995	.996	.051 [.039, .063]	−370.42		–	–	–	–

***Note*.** HeartQOL-BM= Heart Quality-of-Life-Bahasa Malaysia questionnaire; HQ = HeartQoL item; *df* = degrees of freedom; SBχ^2^ = Satorra–Bentler corrected maximum likelihood chi-square; TLI_robust_ = corrected Tucker–Lewis Index; CFI_robust_ = corrected comparative fit index; CAIC_robust_ = corrected, consistent Akaike information criterion; RMSEA_robust_ = corrected root mean square error of approximation; CR = composite reliability; AVE = average variance extracted; MSV = maximum shared squared variance; ASV = average shared squared variance.

^a^Hu & Bentler (1999). ^b^MacCallum et al. (1996). ^c^ Factor loadings at ≥ .80 with an F1–F2 correlation of .64. ^d^De Smedt et al. (2016). ^e^ Values did not meet criteria as F2 was highly correlated with F3 at .93. ^f^ First-order factor loadings at ≥ .64 with F1 and F2 loadings at .69 and .60, respectively.

**Table 4.** Tests for Invariance of the HeartQoL-BM Model Across Subgroups: Summary of Goodness-of-Fit Statistics in Multiple-Group Analyses

Index	MLχ^2^	SB χ^2^	*df*	CFI_robust_ (≥ .95 = good fit^ a^)	RMSEA_robust_ [90% CI] (< .06 = good fit^ a^; > .10 = poor fit^ b^)		Model Comparison		∆SB χ^2^		∆*df*	*p* (> .05)	∆CFI_robust_ (≤ .01)
		(Criteria of good fit)							(Criteria of invariance)				
Native BM speakers (*n* = 190) vs. nonnative BM speakers (*n* = 183)													
Model 1: configural model (no parameter constraints between groups)													
	639.34	237.15	152	.995	.055	[.041, .068]		–		–	–	–	–
Model 2: measurement model invariant (factor loadings between groups equally constrained)													
	645.03	242.50	164	.995	.051	[.036, .064]		2 vs. 1	2.582		12	> .1	< .001
Model 3: structural model invariant (all factor loadings and factor covariances between groups equally constrained)													
	651.66	245.24	165	.995	.051	[.037, .064]		3 vs. 1	5.587		13	> .1	< .001
Myocardial infarction (*n* = 123) vs. angina (*n* = 139) vs. heart failure (*n* = 111)													
Model 4: configural model (no parameter constraints between groups)													
	722.85	321.95	228	.993	.058	[.042, .072]		–	–		–	–	–
Model 5: measurement model invariant (factor loadings between groups equally constrained)													
	760.13	341.52	252	.993	.054	[.038, .067]		5 vs. 4	18.272		24	> .1	< .001
Model 6: Structural model invariant (all factor loadings and factor covariances between groups equally constrained)													
	771.41	340.23	254	.993	.052	[.037, .066]		6 vs. 4	19.733		26	> .1	< .001

***Note*.** HeartQOL-BM = Heart Quality-of-Life (HeartQoL)-Bahasa Malaysia questionnaire; MLχ^2^ = maximum likelihood chi-square; SBχ^2^ = Satorra–Bentler scaled chi-square; *df* = degrees of freedom; ∆ = difference; CFI_robust_ = corrected comparative fit index; RMSEA_robust_ = corrected root mean square error of approximation.

^a^Hu & Bentler (1999). ^b^ MacCallum et al. (1996).

## Discussion

The perspectives of participants, which were gathered via cognitive interviews, were congruent with the assessments of the sociolinguists, suggesting that the two methods are mutually complementary. Noteworthy findings are elaborated in this section to show the value of qualitative inquiry to eliciting insights and identifying potential gaps. In addition, how the resultant version achieved the six types of equivalence is discussed to support the efficacy of applying the cognition-and-equivalence model, using cognitive interviews with or without expert assessment, to pretest translated questionnaires.

### Noteworthy Observations From the Cognitive Interviews and Expert Assessments

In this study, by translation alone, neither the FB nor DP method achieved optimal equivalence. Moreover, qualitative pretesting methods such as modified cognitive interviewing may offer additional insight. Although most items exhibited good semantic equivalence (X_GM_ > 4.0), the findings from the experts and the cognitive interviews suggest that minor revisions should be made to enhance equivalence. For example, adding alternative wordings to items (e.g., HQ7, HQ10, HQ12) may promote comprehension of item meanings among respondents (Weeks et al., 2007). The cognitive interviews highlighted potential gaps of equivalence, including gaps overlooked by translators. For example, the technical term “physical” (“fizikal”) in both the FB and DP versions of HQ7 was found to be potentially problematic for respondents with lower levels of education. Conversely, the cognitive interviews tended to affirm the suggestions of the translators. For example, adding an example of a football field to item HQ4_DP_ helped respondents better comprehend the distance covered by 100 yards. However, as this example was not included in the original item, HQ4_DP_ earned a lower equivalence rating from the experts (HQ4_DP_ = X_GM_ of 4.05 vs. HQ4_FB_ = X_GM_ of 4.78). The neutrality of sociolinguistic experts enhanced objectivity in the translation assessments and played a supplementary role in cognitive interviews by aiding healthcare researchers who often lack expertise regarding addressing cross-cultural issues, item structural problems, and culture-related linguistic stylings (Conway et al., 2014).

Furthermore, in this study, a potential drawback in terms of overemphasizing back-translation was observed in HQ6_FB_. This overemphasis was rectified to address a concern raised on the nonalike wording between back-translated and source versions, although these words were equivalent in meaning. To produce an exact back-translated wording for the term “short of breath,” the revision of HQ6_FB_ resulted in the unnatural sounding term “nafas pendek,” which was rated poorly (X_GM_ = 3.11). This example illustrates the limits of back-translation, especially when words do not have equivalent expressions in other languages and when grammatical structures differ between languages, which may convey an equivalent meaning but use different wordings in the back-translated and source texts (Tsai et al., 2018). Therefore, back-translation should be used to promote discussion rather than provide an absolute indicator of quality. A competent bilingual committee that is carefully selected should be able to derive and assess semantic equivalence in translation (Epstein et al., 2015) and to identify item content that differs by culture. In this study, the suggestion made by the better-educated respondents to add local household chores (sweeping and mopping) to item HQ2_DP_ in the DP method resulted in better item equivalence during cognitive interviews and expert assessments. In addition to affirming the relevance of local chores, the cognitive interviews revealed the different values held among the participants, with some viewing household chores as “a woman's job,” which is common in cultures with patriarchal family systems. This should be a point of consideration for those working to develop or cross-culturally adapt questionnaire items in the future.

According to Herdman et al. (1998), translation should consider using appropriate language levels, including the lingo and syntax of common language, to meet the needs of the target population. Hence, the process of translating the HeartQoL-BM version considered the variance in BM proficiency within Malaysia's multiethnic society and the country's unique linguistic landscape in which code-switching and code-mixing between English and BM are practiced commonly in social conversation (Y. L. Lee et al., 2012). Findings from the cognitive interviews and experts supported using lay lingo such as “tension,” “frust,” and “susah hati” for respective items HQ9, HQ11, and HQ12 to clarify meaning and more accurately reflect the original texts (Herdman et al., 1998). One final, notable encounter in this study was the initial misunderstanding of researchers regarding the original developer's intent for HQ14_DP_. Hence, a document or manual that explicitly explains the conceptual basis of each item would be useful to facilitate the interpretation of item intent by translators (Wild et al., 2005).

### The Six Equivalence Types of the Final Heart Quality-of-Life–Bahasa Malaysia

The semantic and item equivalences of the HeartQoL-BM were substantiated in the analysis of cognitive interview data using the cognition-and-equivalence model. In addition, areas of potential gaps because of differences in sociocultural factors, educational level, and linguistic expression were also identified. These findings, together with the opinions of the experts, offer valuable insights that may be used to enhance semantic and item equivalence in the final version of the HeartQoL-BM.

The conceptual, item, measurement, and operational equivalences of HeartQoL-BM were substantiated in the CFA. The two-factor Model B (Table 3) confirmed that HeartQoL-BM had adequate conceptual and item equivalences because the four items in the emotional dimension and the 10 items in the physical dimension respectively formed the single, underlying structures of F1 and F2, as discussed in the source study of De Smedt et al. (2016). Model B was shown to have good fit (i.e., a Tucker–Lewis index of .995, a comparative fit index of .996, and a root mean square error of approximation of .051). Moreover, adequate convergent validity among items was found in the physical and emotional subscales, with respective composite reliability scores of .94 and .84 and respective average variance extractions of .83 and .72. Findings for the model fit and internal consistency reliability of the HeartQoL-BM were comparable with the source (De Smedt et al., 2016; Oldridge et al., 2014), supporting measurement equivalence. Using a method of administration similar to source studies (De Smedt et al., 2016; Oldridge et al., 2014), the HeartQoL-BM elicited a comparable scoring distribution that confirmed operational equivalence. As shown in Table 2, all ceiling and floor effects were considered negligible, except for the emotional subscale, which showed a high ceiling effect > 15% among patients with angina and myocardial infarction.

The functional equivalence of HeartQoL-BM was confirmed in this study, with cognitive interviews findings supportive of semantic and item equivalence and confirmation of conceptual, item, measurement, and operational equivalences in the CFA. Therefore, studies that use HeartQoL-BM may be expected to yield comparable results with studies that use the original HeartQoL. Moreover, in this study, CFAs of the second-order model and factorial invariance testing were conducted to provide information that were precluded in the source study (De Smedt et al., 2016). As shown in Table 3, the fit indices for Model B.1 were good, thus supporting the validity of global scale scores in representing overall HRQoL level. The tests of invariance shown in Table 4 support the use of HeartQoL-BM for both native and nonnative BM speakers and using HeartQoL-BM to measure HRQoL in patients with IHD experiencing angina, myocardial infarction, or heart failure.

### Study Limitations

Although more DP items than FB items constitute the final HeartQoL-BM, the DP method is not necessarily a better translation approach, as the sample of experts used in this study was small. Moreover, the qualitative nature of cognitive interviewing and the decision making in item finalization are inherently subjective. However, the feedback from experts and cognitive interviews provided important insights that were used to adapt the questionnaire to the needs and cultural expectations of the targeted patient population, while adhering to copyright and licensing agreement requirements. Invariances across gender and educational level were not tested, as the female–male gender ratio was ≈1:5 and the tertiary–secondary educational level ratio was ≈1:3. These unbalanced ratios within the sample group had different impacts on the χ^2^ of the configural model, which may lead to invalid results.

### Conclusions

The complementary and congruent findings of the expert assessment and cognitive interviews confirmed semantic and item equivalences, highlighted potential gaps to be addressed/rectified, and aided decision making with regard to item finalization. Findings from the CFA of the HeartQoL-BM confirmed its conceptual, item, measurement, and operational equivalences to the original instrument. Confirmation of these five equivalence types adequately shows the functional equivalence of the HeartQoL-BM to the source instrument. Therefore, the HeartQoL-BM is a potentially valid and acceptable core IHD-specific HRQoL instrument that functions equivalently across Malaysia's multiethnic culture and across those who speak Malay as a mother tongue and those who speak Malay as a second language. These findings lend support to adapting Herdman et al.'s and Collins' models (e.g., cognition-and-equivalence model) to the cross-cultural adaptation and translation of questionnaires.
